# Risk of cardiac-related death in astrocytoma patients treated with chemotherapy: A competing risk analysis using the SEER database

**DOI:** 10.3389/fcvm.2023.996354

**Published:** 2023-04-25

**Authors:** Xuezhen Wang, Xiaoxia Li, Yufan Wu, Jinsheng Hong, Dajun Chai, Mingwei Zhang

**Affiliations:** ^1^ Department of Radiotherapy, Cancer Center, The First Affiliated Hospital, Fujian Medical University, Fuzhou, China; ^2^ National Regional Medical Center, Binhai Campus of the First Affiliated Hospital, Fujian Medical University, Fuzhou, China; ^3^ Key Laboratory of Radiation Biology of Fujian Higher Education Institutions, The First Affiliated Hospital, Fujian Medical University, Fuzhou, China; ^4^ Cardiovascular Department, The First Affiliated Hospital, Fujian Medical University, Fuzhou, China

**Keywords:** glioma, chemotherapy, cardiac-related death, competing risk analysis, PSM

## Abstract

**Purpose:**

To explore the impact of chemotherapy on the risk of cardiac-related death in astrocytoma patients.

**Methods:**

We retrospectively evaluated astrocytoma patients diagnosed between 1,975 and 2016 in the Surveillance, Epidemiology, and End Results (SEER) database. Using Cox proportional hazards models, we compared the risks of cardiac-related death between a chemotherapy group and non-chemotherapy group. Competing-risks regression analyses were used to evaluate the difference in cardiac-related death. Also, propensity score matching (PSM) was employed to reduce confounding bias. The robustness of these findings was evaluated by sensitivity analysis, and E values were calculated.

**Results:**

A total of 14,834 patients diagnosed with astrocytoma were included. Chemotherapy (HR = 0.625, 95%CI: 0.444–0.881) was associated with cardiac-related death in univariate Cox regression analysis. Chemotherapy was an independent prognostic factor for a lower risk of cardiac-related death before (HR = 0.579, 95%CI: 0.409–0.82, *P* = 0.002) and after PSM (HR = 0.550, 95%CI: 0.367–0.823 *P* = 0.004). Sensitivity analysis determined that the E-value of chemotherapy was 2.848 and 3.038 before and after PSM.

**Conclusions:**

Chemotherapy did not increase the risk of cardiac-related death in astrocytoma patients. This study highlights that cardio–oncology teams should provide comprehensive care and long-term monitoring for cancer patients, especially those with an increased risk of cardiovascular disease.

## 1. Introduction

Gliomas are the most common primary tumors of the brain. They account for more than half of all primary tumors of the central nervous system (CNS). The World Health Organization classification of tumors of the central nervous system (CNS) (5th Edition) (1) was published in 2021, but the choice of treatment was based on the previous criteria of tumor diagnosis.

The standard treatment for glioma is resection with or without radiotherapy and/or chemotherapy (2, 3). Chemotherapy can prolong the progression-free survival and overall survival (OS) of glioma patients (3). In cases where glioma recurs after standard treatment, there is currently no standard chemotherapy regimen available, and instead an individualized approach is typically employed. Therefore, careful consideration of the benefits and drawbacks of chemotherapy is crucial (4).

Antineoplastic therapy, such as chemotherapy, is known to prolong the survival of cancer patients. However, it can also induce cardiotoxicity, including cardiomyopathy, arrhythmias and other types of cardiovascular disease (CVD) (5, 6). CVD has become the second leading cause of death in patients with CNS tumors (7–9). A study on the Wales-based Secure Anonymous Information Linkage (SAIL) and US Surveillance, Epidemiology, and End Results (SEER) databases showed that the standardized mortality ratio (SMR) for CVD in patients with gliomas was 2.24 and 1.98, respectively (8).

However, evidence that clinical, imaging or laboratory parameters could identify astrocytoma patients at a higher cardiac risk is lacking. Studies on the risk of CVD or mortality for people with a glioma have reported overall findings without revealing histologic subtypes. This scenario may mask tumor heterogeneity, which could lead to different impacts on CVD mortality (8). Additionally, previous studies have focused on the effect of chemotherapy on all-cause mortality, with little attention given to the effect of chemotherapy on cardiac-related death in patients with astrocytoma. A better understanding of the risk factors associated with cardiac-related death and risk stratification in astrocytoma could help facilitate targeted interventions to lengthen survival.

Our study aimed to explore whether chemotherapy increases the risk of cardiac-related death in patients with astrocytoma based on a cohort from the SEER database. We used competing-risks regression analysis and sensitivity analysis. Our findings could aid clinicians in making informed treatment decisions by providing a better understanding of the risks associated with chemotherapy, as well as enabling more precise predictions of outcome in patients with astrocytoma.

## 2. Materials and methods

### 2.1. Study population

This retrospective study was undertaken with data from the SEER database using SEER*Stat 8.3.8. Our selected data source was the “Incidence—SEER 18 Regs Research Data + Hurricane Katrina Impacted Louisiana Cases, Nov 2018 Sub (1975–2016 varying)” database.

The inclusion criteria were: (1) the primary site of the tumor was the brain; (2) the histologic type was based on the International Classification of Diseases, 3rd edition (ICD-O-3), including 9400/3 [astrocytoma, not otherwise specified (NOS)], 9410/3 (protoplasmic astrocytoma), 9410/3 (protoplasmic astrocytoma) or 9420/3 (fibrillary astrocytoma); (3) the year of the diagnosis was between 1975 and 2016.

The exclusion criteria were: (1) unknown survival time; (2) missing important clinical data (e.g., age, sex, race, primary site of tumor and cause of death).

### 2.2. Selection of variables

The study variables provided by the SEER database were: demographic variable (“age at diagnosis”, “sex”, “race”, “marital status at diagnosis”, “year of the diagnosis”), clinicopathologic variables (“primary site”, “histologic type ICD-O-3”, “tumor size”, “sequence number”), treatment status (“radiation recode”, “chemotherapy recode”, “surgery record”, “surgical method”) and survival status (“survival months”, “SEER cause-specific death classification”, “SEER other cause of death classification”).

### 2.3. Endpoint definition

Cardiac-related death was the primary endpoint. Deaths resulting from accidents or diseases other than cardiac-related death was considered to be the competing risks. The data of cardiac-related death and non-cardiac-related death were extracted from the variables “SEER cause-specific death classification” and “SEER other cause of death classification” in the SEER database (https://seer.cancer.gov/causespecific/).

### 2.4. PSM

Propensity-score matching (PSM) was done using the nearest-neighbor-matching method with a caliper of 0.1 on the propensity scale with logistic regression. PSM was undertaken to match different variables between patients who underwent chemotherapy vs. patients who did not undergo chemotherapy. The variables were age, sex, race, marital status, year of diagnosis, primary site of tumor, histologic type of tumor, radiotherapy and surgery, and we used a 1:1 ratio for patients.

### 2.5. Statistical analyses

R 4.0.1 (R Foundation for Statistical Computing, Vienna, Austria; www.r-project.org/) was used for statistical analyses. Statistical analyses of categorical data were done using the chi-square test. “Cardiac-related death” was defined as any death caused by a heart disease. To gain a first insight into the relationship between chemotherapy and cardiac-related disease-specific survival, Kaplan–Meier survival curves were estimated and compared by the log-rank test. The association between chemotherapy and the risk of cardiac-related death was assessed by univariable and multivariable Cox proportional hazards regression models. The competing risks model was employed with a cumulative incidence function (CIF) to estimate the cumulative probability of cardiac-related death and non-cardiac-related death. The CIF was compared among subgroups by the Gray test. The Fine–Gray proportional hazards model was employed to analyze competing risks using “cmprsk” within R. Adopt E-value (10) was used to evaluate the extent of unmeasured confounding factors that could have influenced the results. *P* < 0.05 (two-sided) was considered significant.

## 3. Results

### 3.1. Patient characteristics before PSM

A total of 14,834 patients diagnosed with astrocytoma before 2016 were identified. Patients were divided into a chemotherapy group (*n* = 3832) and non-chemotherapy group (*n* = 11,002). The median duration of survival among the whole group was 25 [interquartile range (IQR): 7–96] months. The median duration of survival in the chemotherapy group was 18  (IQR: 8–52) months, and that in non-chemotherapy group was 30 (IQR: 7–114.75) months. The demographic and clinicopathological data of the two groups are shown in Table 1.

**Table 1:** Characteristics of patients before and after PSM.

Variables	Before PSM			After PSM		
	Chemotherapy NO (*n* = 11,002)	Chemotherapy YES (*n* = 3,832)	*P*	Chemotherapy NO (*n* = 3,350)	Chemotherapy YES (*n* = 3,349)	*P*
Age, *n* (%)			<0.001			0.482
∼30	3,166 (29)	748 (20)		683 (20)	715 (21)	
31–50	3,067 (28)	1,248 (33)		1,078 (32)	1,026 (31)	
51–70	3,057 (28)	1,500 (39)		1,272 (38)	1,274 (38)	
71∼	1,712 (16)	336 (9)		317 (9)	334 (10)	
Sex, *n* (%)			<0.001			0.034
Female	4,978 (45)	1,574 (41)		1,435 (43)	1,348 (40)	
Male	6,024 (55)	2,258 (59)		1,915 (57)	2,001 (60)	
Race, *n* (%)			0.101			0.331
Black	758 (7)	232 (6)		238 (7)	227 (7)	
Others	528 (5)	204 (5)		164 (5)	190 (6)	
White	9,716 (88)	3,396 (89)		2,948 (88)	2,932 (88)	
Marital_status, *n* (%)			<0.001			0.094
Divorced/separated	765 (7)	286 (7)		231 (7)	273 (8)	
Married	5,549 (50)	2,357 (62)		2,034 (61)	1,949 (58)	
Single/Unmarried	3,497 (32)	938 (24)		858 (26)	881 (26)	
Widowed/Others	1,191 (11)	251 (7)		227 (7)	246 (7)	
Diagnosis, *n* (%)			<0.001			0.268
∼2005	8,252 (75)	2,001 (52)		2,041 (61)	1,995 (60)	
2005–2016	2,750 (25)	1,831 (48)		1,309 (39)	1,354 (40)	
Grade, *n* (%)			<0.001			<0.001
1	830 (8)	104 (3)		197 (6)	92 (3)	
2	3,426 (31)	609 (16)		962 (29)	548 (16)	
3	2,016 (18)	1,011 (26)		577 (17)	945 (28)	
4	1,199 (11)	898 (23)		374 (11)	768 (23)	
Unknown	3,531 (32)	1,210 (32)		1,240 (37)	996 (30)	
Primary.Site, *n* (%)			<0.001			0.373
Infratentorial	1,399 (13)	275 (7)		255 (8)	271 (8)	
Others	2,557 (23)	870 (23)		789 (24)	826 (25)	
Supratentorial	7,046 (64)	2,687 (70)		2,306 (69)	2,252 (67)	
Hist.Type, *n* (%)			<0.001			0.592
Fibrillary astrocytoma	1,035 (9)	570 (15)		430 (13)	430 (13)	
Gemistocytic astrocytoma	543 (5)	421 (11)		338 (10)	317 (9)	
NOS	9,326 (85)	2,827 (74)		2,574 (77)	2,589 (77)	
Protoplasmic astrocytoma	98 (1)	14 (0)		8 (0)	13 (0)	
Tumor_Size, *n* (%)			<0.001			0.464
∼4	1,141 (10)	606 (16)		466 (14)	452 (13)	
4∼	792 (7)	600 (16)		394 (12)	426 (13)	
Unknown	9,069 (82)	2,626 (69)		2,490 (74)	2,471 (74)	
Surgery, *n* (%)			<0.001			0.108
NO	1,801 (16)	871 (23)		842 (25)	847 (25)	
Unknown	6,276 (57)	1,424 (37)		1,500 (45)	1,423 (42)	
YES	2,925 (27)	1,537 (40)		1,008 (30)	1,079 (32)	
Surgery. Method, *n* (%)			<0.001			0.01
Biopsy&Local excision	851 (8)	457 (12)		311 (9)	332 (10)	
Gross total resection	1,132 (10)	469 (12)		337 (10)	301 (9)	
No surgery	1,801 (16)	871 (23)		842 (25)	847 (25)	
Others	6,352 (58)	1,451 (38)		1,519 (45)	1,446 (43)	
Subtotal resection	866 (8)	584 (15)		341 (10)	423 (13)	
Radiation, *n* (%)			<0.001			0.65
NO	7,357 (67)	1,593 (42)		1,591 (47)	1,571 (47)	
YES	3,645 (33)	2,239 (58)		1,759 (53)	1,778 (53)	
Sequence. Number, *n* (%)			0.145			0.816
NO	10,281 (93)	3,553 (93)		3,104 (93)	3,109 (93)	
YES	721 (7)	278 (7)		246 (7)	240 (7)	

Note: Sequence number, previous history of malignancy.

### 3.2. Patient characteristics after PSM

PSM was conducted to minimize possible confounding effects. We discovered that 3,349 pairs of patients who underwent chemotherapy vs. those who did not receive chemotherapy were matched by a 1:1 ratio, respectively. The standard mean difference of all parameters after matching was <0.1 (Supplementary Figure S1). No significant differences in characteristics at baseline were observed in the two cohorts except for sex, grade and surgery method (Table 1).

### 3.3. Survival analyses

**Figure 1:**
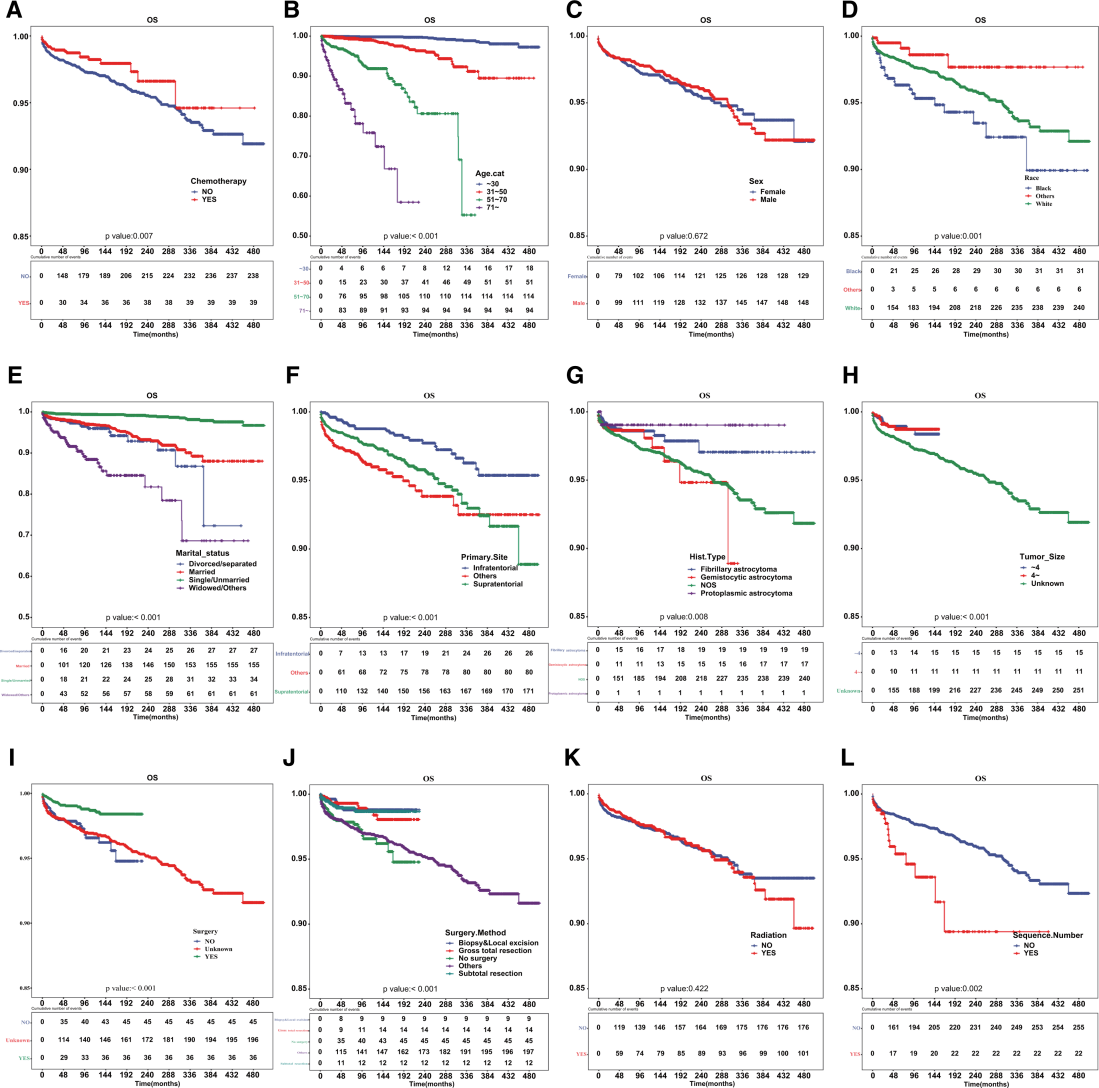
Cardiac disease-specific survival curve according to patient characteristics. Cardiac disease-specific survival curve of (**A**) chemotherapy, (**B**) age, (**C**) sex, (**D**) race, (**E**) marital status, (**F**) primary site of tumor, (**G**) histologic type, (**H**) tumor size, (**I**) surgery, (**J**) surgical method, (**K**) radiotherapy and (**L**) sequence number.

Patients who underwent chemotherapy had a higher prevalence of cardiac-related disease-specific survival compared to those who did not receive chemotherapy (Figure 1A). A univariate Cox regression model was established to identify potential prognostic factors. Our findings showed that chemotherapy (hazard ratio [HR] = 0.625, 95% confidence interval [CI]: 0.444–0.881), age (31–50 vs. ≤30 years: HR = 6.564, 95%CI: 3.743–11.51; 51–70 vs. ≤30 years: HR = 41.38, 95%CI: 23.775–72.021; ≥71 vs. ≤ 30 years: HR = 161.748, 95%CI: 90.175–290.13), race (others *vs*. black: HR = 0.266, 95%CI: 0.111–0.638; white *vs*. black: HR = 0.627, 95%CI: 0.432–0.912), marital status (single/unmarried *vs*. divorced/separated: HR = 0.147, 95%CI: 0.088–0.245; widowed/others *vs*. divorced/separated: HR = 2.482, 95%CI: 1.575–3.91), primary site (HR = 1.985, 95%CI: 1.305–3.02), surgery (HR = 0.388, 95%CI: 0.25–0.601) and sequence number (HR = 1.961, 95%CI: 1.263–3.043) were associated with cardiac-related death (Table 2). However, upon multivariable Cox regression analysis, chemotherapy was not an independent factor for patients with astrocytoma.

**Table 2:** Univariate and multivariate Cox regression analysis.

	Univariate analysis		Multivariate analysis	
	HR (95%CI)	*P*	HR (95%CI)	*P*
**Age. cat**				
31–50 vs. ∼30	6.564 (3.743–11.51)	<0.001	5.989 (3.136–11.434)	<0.001
51–70 vs. ∼30	41.38 (23.775–72.021)	<0.001	37.376 (19.438–71.866)	<0.001
71∼ vs. ∼30	161.748 (90.175–290.13)	<0.001	130.233 (64.925-261.234)	<0.001
**Sex**				
Male vs. female	0.95 (0.75–1.204)	0.672	1.24 (0.969–1.588)	0.088
**Race**				
Others vs. black	0.266 (0.111–0.638)	0.003	0.192 (0.08–0.462)	<0.001
White vs. black	0.627 (0.432–0.912)	0.015	0.41 (0.28–0.599)	<0.001
**Marital status**				
Married vs. divorced/separated	0.776 (0.515–1.168)	0.224	0.718 (0.474–1.086)	0.117
Single/unmarried vs. divorced/separated	0.147 (0.088–0.245)	<0.001	0.599 (0.34–1.056)	0.077
Widowed/others vs. divorced/separated	2.482 (1.575–3.91)	<0.001	1.098 (0.684–1.765)	0.698
**Primary site of tumor**				
Others vs. infratentorial	2.936 (1.876–4.595)	<0.001	1.247 (0.783–1.985)	0.352
Supratentorial vs. infratentorial	1.985 (1.305–3.02)	0.001	0.997 (0.645–1.542)	0.991
**Histologic type**				
Gemistocytic astrocytoma vs. fibrillary astrocytoma	1.731 (0.9–3.332)	0.1	1.537 (0.794–2.976)	0.202
NOS vs. fibrillary astrocytoma	1.732 (1.086–2.765)	0.021	1.246 (0.778–1.996)	0.36
Protoplasmic astrocytoma vs. fibrillary astrocytoma	0.523 (0.07–3.91)	0.528	0.587 (0.078–4.405)	0.604
**Tumor size**				
4∼ vs. ∼4	0.96 (0.441–2.091)	0.919	1.018 (0.466–2.22)	0.965
Unknown vs. ∼4	2.026 (1.198–3.426)	0.008	1.389 (0.782–2.469)	0.263
**Surgery**				
Unknown vs. NO	1.034 (0.741–1.443)	0.843	1.309 (0.895–1.914)	0.165
YES vs. NO	0.388 (0.25–0.601)	<0.001	0.654 (0.411–1.042)	0.074
**Radiotherapy**				
YES vs. NO	0.904 (0.708–1.156)	0.422	1.005 (0.765–1.32)	0.971
**Chemotherapy**				
YES vs. NO	0.625 (0.444–0.881)	0.007	0.703 (0.491–1.006)	0.054
**Sequence number**				
YES vs. NO	1.961 (1.263–3.043)	0.003	0.857(0.549–1.337)	0.496

Note: Sequence number, previous history of malignancy.

**Figure 2:**
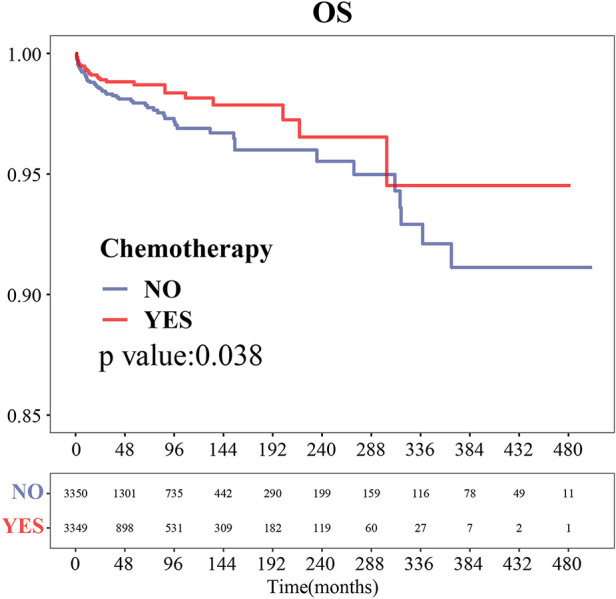
Cardiac disease-specific survival curve of chemotherapy after PSM.

After PSM, the cardiac-related disease-specific survival outcome between the chemotherapy group and non-chemotherapy group was the same, with a *P*-value of 0.038 (Figure 2).

### 3.4. Competing-risks regression analysis before PSM

**Figure 3:**
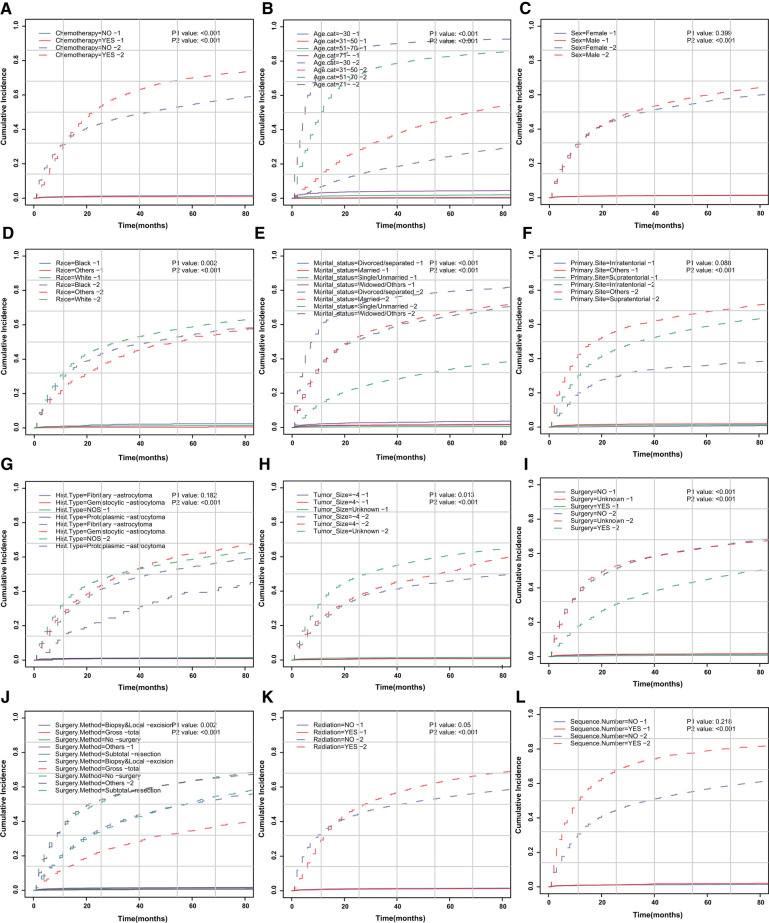
CIF curve of death according to patient characteristics. CIF curve of (**A**) chemotherapy, (**B**) age, (**C**) sex, (**D**) race, (**E**) marital status, (**F**) primary site of tumor, (**G**) histologic type, (**H**) tumor size, (**I**) surgery, (**J**) surgical method, (**K**) radiotherapy and (**L**) sequence number.

**Figure 4:**
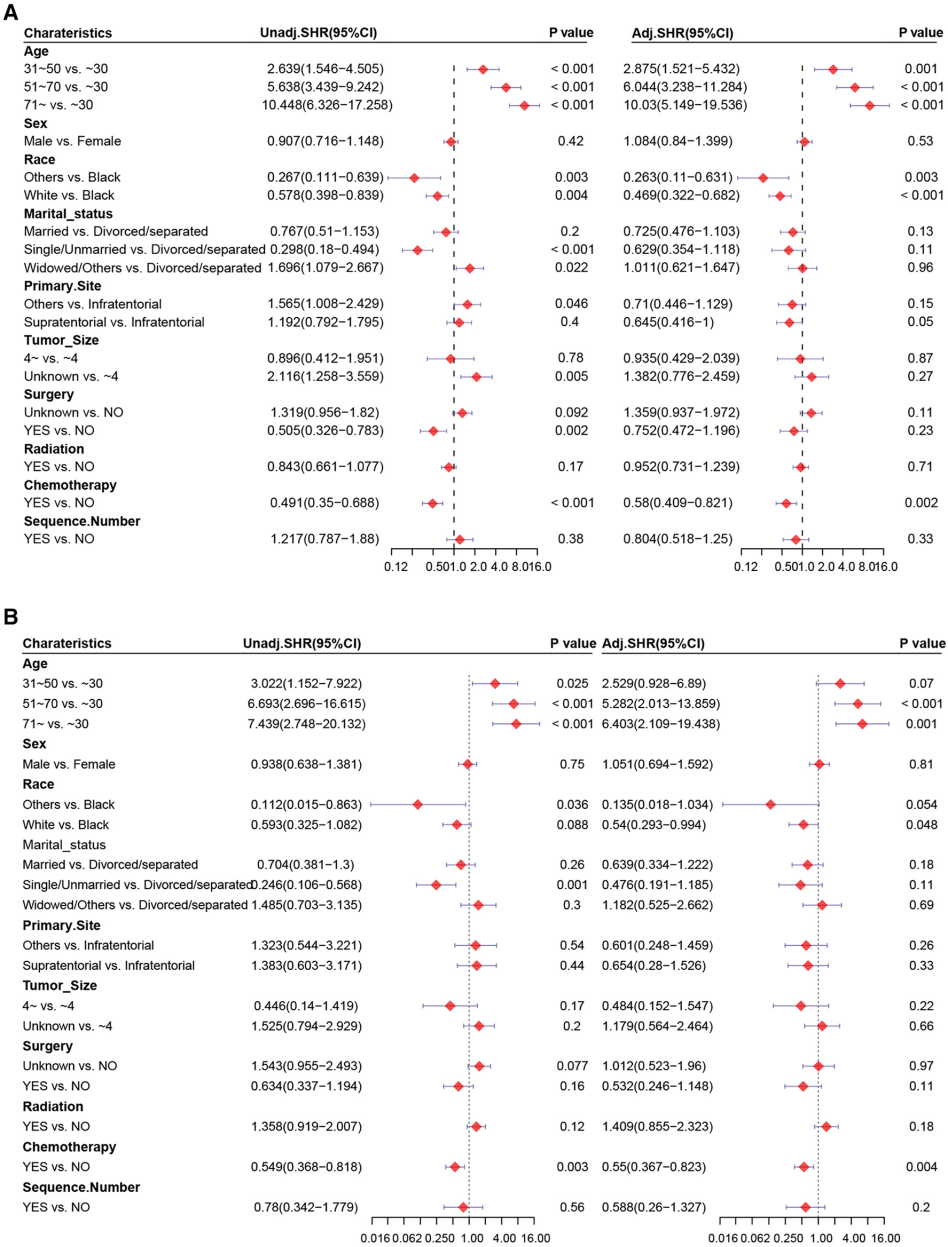
Forest plots of univariate and multivariate Cox regression analysis. Univariate and multivariate Cox regression analysis of chemotherapy (**A**) before and (**B**) after PSM.

We aimed to address the issue of competing risk bias, so we utilized the Fine–Gray model to evaluate the risks of independent prognostic variables with non-cardiac-related death as a competing risk. Among the 10,602 deaths events, 277 were attributed to cardiac-related death. The cumulative incidence of cardiac-related death and non-cardiac-related death is demonstrated in Figure 3. The univariate analysis results are displayed in Figure 4A. It is found that chemotherapy was associated with a lower risk of cardiac-related death (HR = 0.491, 95%CI: 0.35–0.688, *P *< 0.001). The multivariate competing-risks proportional hazards model included the same covariates as the univariate analysis. Chemotherapy remained significant in the multivariate analysis (HR = 0.579, 95%CI: 0.409–0.821, *P* = 0.002) (Figure 4A). Subgroups of age (51–70 or ≥71 years), sex (male or female), race (white), marital status (married), diagnosis (before 2005), primary site (supratentorial or others), tumor size (unknown), surgery (no or unknown), surgical method (no surgery or others), radiotherapy (yes or no) and sequence number (yes or no) demonstrated a reduction in the risk of cardiac-related death with chemotherapy. There was no significant interaction between chemotherapy and other study variables (*P*_interaction _> 0.05) (Supplementary Figure S2A).

### 3.5. Competing-risks regression analysis after PSM

PSM was carried out to eliminate the imbalance of baseline characteristics between groups. After PSM, results based on the competing risk model demonstrated that chemotherapy was associated significantly with cardiac-related death (HR = 0.549, 95%CI: 0.368–0.818, *P* = 0.003) and was an independent prognostic factor of a lower risk of cardiac-related death (HR = 0.55, 95%CI: 0.367–0.823, *P* = 0.004) (Figure 4B). The subgroups of sex (male), marital status (married), diagnosis (before 2005), primary site (supratentorial), tumor size (unknown), surgery (unknown), radiotherapy and no sequence number were demonstrated to reduce the risk of cardiac-related death with chemotherapy. No significant interaction between chemotherapy and those variables was found(*P*_interaction _> 0.05) (Supplementary Figure S2B).

### 3.6. Sensitivity analysis

Before PSM, it was found that the relative risk (RR) of death in the chemotherapy group was 0.579, and the E-value (95%CI) was determined to be 2.848 (1.737–4.325) (Supplementary Figure S3A). After PSM, the RR was 0.55 and the E-value (95%CI) was determined to be 3.038 (1.726–4.893) for death in the chemotherapy group (Supplementary Figure S3B).

## 4. Discussion

Cancer treatment-related cardiotoxicity has gained increasing attention recently. Chemotherapy plays an important role in the treatment of patients with high-risk, low-grade gliomas (11, 12). However, the evidence regarding whether chemotherapy increases the risk of cardiac-related death in patients with astrocytoma is not yet conclusive.

We investigated the impact of chemotherapy on cardiac-related death in patients with astrocytoma based on clinicopathological characteristics and the survival data of 14,834 patients with astrocytoma in the SEER database. With cardiac-related death as the endpoint, the Kaplan–Meier curve showed longer cardiac-related disease-specific survival in patients receiving chemotherapy than those who did not. However, a correlation between chemotherapy and cardiac-related death was not found upon multivariable Cox regression analysis. As retrospective observational studies cannot eliminate the influence of confounding factors on the outcome through randomization, we conducted PSM to achieve the effect of “post-randomization”. Before and after PSM, patients who received chemotherapy had significantly longer cardiac-related disease-specific survival compared with those who did not receive chemotherapy. The competing risk regression model showed an independent prognostic role of chemotherapy for cardiac-related death. Furthermore, E-values were calculated in order to evaluate the influence of unmeasured confounding factors. The E-value of chemotherapy was 2.848 and 3.038 before and after PSM indicating that an unmeasured confounding factor exhibiting an extremely powerful association strength is required to overturn the obtained results. However, this type of factor is almost non-existent in this study.

One strength of our study is that it relates the risk of cardiac-related death in astrocytoma patients to whether they received chemotherapy or not during the disease course. Another strength of our study is that it was population-based, involving all patients with astrocytoma in the SEER database who had or did not have chemotherapy during the study period, thereby increasing the generalizability of our findings. Also, the cohort size was large, which aided robust statistical analyses. Our study also avoided the tendency in randomized clinical trials to omit patients in poor health. We minimized the potential for bias caused by a lack of information by only enrolling patients for whom detailed information on chemotherapy was available. However, because it was a retrospective study, we cannot rule out the possibility of confounding factors that may have influenced our results. To mitigate this, we used PSM for post-randomization to reduce the influence of confounding factors. In addition, E-value was used to assess the effect of unknown confounding factors on the results to evaluate the robustness of these findings. No prospective clinical studies have explored the correlation between chemotherapy and cardiac-related death in astrocytoma patients, so our results based on a large cohort after PSM provide valuable guidance (13).

CVD is an important cause of death in cancer survivors (7). Many studies have discussed the risk of antineoplastic therapy-related cardiac diseases among survivors of breast cancer (14), lung cancer (15), and hematologic malignancies (15), but the risk of cardiac disease in patients with astrocytoma is not known.

The study conducted by Matthews and colleagues, which included 1,005 postmenopausal breast cancer patients in the UK and 22,027 patients in the USA, found that women treated with aromatase inhibitors showed a higher risk of several cardiovascular outcomes compared with those in users of tamoxifen. A study by Sun (15) indicated that of all competing causes of death in non-small-cell lung cancer, CVD resulted in the highest cumulative mortality (with the exception of lung cancer). Chemotherapy is considered to be a risk factor for CVD in cancer patients (14, 16). Platinum-based chemotherapy is a cardiovascular risk factor for survivors of testicular cancer (17), mainly due to drug toxicity. In the competing risk regression model, patients receiving chemotherapy (HR = 0.55, 95%CI: 0.367–0.823) had a lower risk of cardiac-related death than others, which is consistent with previous findings (15). In the absence of specific chemotherapy regimens, it is difficult to determine whether this effect is related to drug toxicity is not possible because clinicians may have assessed the potential combined risk before chemotherapy so that patients with high risk of CVD are excluded from treatment due to contraindications to chemotherapy. Patients with good cardiac function, excellent physical status and few contraindications to chemotherapy are more likely to receive chemotherapy, which could also influence the results.

A better understanding of the risk factors contributing to cardiac disease in cancer patients can aid in preventing and reducing avoidable morbidity and mortality, ultimately improving prognosis. Factors associated with CVD mortality in the CNS cancers include age, sex, ethnicity, economic deprivation, and no surgical procedure (8). One study focusing on cardiac mortality among 200,000 cancer patients aged 15–39 years who survived 5 years indicated that the number of cardiac-related deaths expected was 1.4-times greater than that of CNS tumors survivors. Additionally, the study suggested that the age at diagnosis plays a crucial role in determining the risk of subsequent cardiac-related death (7). Stratified analysis based on sex revealed that male patients with malignant glioma had a CVD mortality rate of over twofold higher in the SAIL (SMR = 2.66, 95%CI = 1.77–3.82) and SEER (SMR = 2.09, 95%CI = 1.89–2.31) databases compared to female patients (8). In our study, the HR for cardiac-related death in male patients was 0.448 (95%CI: 0.260–0.771). The reason for the discrepancy in values may be due to the inclusion of all histologic types of gliomas in the SAIL and SEER studies (e.g., glioblastoma), whereas we targeted the specific histologic type of astrocytoma. In addition, the risk of cardiotoxicity is associated with the type of chemotherapeutic agent used, as well as other factors such as anthracyclines, anthraquinones, miscellaneous drugs (18), dose (19), hypertension, previous cardiac disease (19), and radiation exposure (20).

For patients at high risk of astrocytoma, the most commonly used chemotherapy regimens are temozolomide (TMZ), procarbazine, lomustine, and vincristine (PCV) (21, 22). The main toxicities of procarbazine and lomustine (CCNU) are myelosuppression and gastrointestinal symptoms, while vincristine is neurotoxic and hepatotoxic, and has been reported to induce ischemic events due to its neurotoxicity (23). Additionally, chronic vincristine treatment may induce cardiovascular toxicity (5). TMZ is a type of oral alkylating agent with the most common side-effects being gastrointestinal and hematologic toxicity (24). Severe hematologic toxicity can lead to life-threatening infections (25), and cardiotoxicity occurs in a small number of patients who experience palpitations and hypertension. It has been reported that alkylating agents can cause direct endothelial damage to cardiomyocytes (26). Given the potential for cardiotoxicity associated with chemotherapeutic agents, the Cardio-Oncology Study Group of the Heart Failure Association of the European Society of Cardiology in collaboration with the International Cardio-Oncology Society Cardiology recommends cardiovascular-risk assessment at baseline for patients scheduled for cancer treatment to minimize the risk of cardiovascular toxicity (27). Meanwhile, the European Society for Medical Oncology consensus also defines cardiovascular toxicity associated with cancer and its treatment, and provides management strategies for prevention, screening, monitoring and treatment of cardiovascular toxicity (28). In the era of “personalized” medicine and the coexistence of multiple treatment modalities for oncology, collaboration between oncology and cardiology departments will facilitate more comprehensive treatment plans for cancer patients.

Our study had five main limitations. Firstly, the lack of detailed information on chemotherapy regimens, doses, frequencies or cycles and radiation dose/fraction in the SEER database prevented a more comprehensive analysis. Secondly, the SEER database does not include records of targeted therapy, immunotherapy and other antineoplastic treatments, the aim of curative or palliative treatment or prognosis-related molecular pathological status which could have influenced the accurate judgment of the prognosis of astrocytoma patients. Thirdly, the survival analysis of subgroups of different types of cardiac disease-related death was not explored due to the variability of heart diseases among patients with cardiac-related disease. Fourthly, the absence of a documented history of previous cardiac disease and assessment of risk factors for cardiac disease (e.g., tobacco smoking, obesity and diabetes mellitus) hampered subgroup analyses. Finally, as this was a retrospective, population-based study, we could only access data from the database up to 2016, which was the entry time for patients with information on adjuvant treatment that could be updated in the database to date. Despite these limitations, our study shed light on the persistent challenge of cardiac-related deaths in astrocytoma patients receiving chemotherapy. These results must be confirmed by further high-level evidence from prospective, multicenter, large-cohort clinical studies.

## 5. Conclusions

We revealed that cardiac-related deaths of astrocytoma patients treated with chemotherapy remain a clinical challenge. Several risk factors for cardiac-related death were identified by using the competing risk regression model. This study highlights that cardio–oncology teams must provide comprehensive care and long-term monitoring for cancer patients, especially those at an increased risk of CVD.

## Data Availability

Publicly available datasets were analyzed in this study. This data can be found here: SEER*Stat 8.3.8, https://seer.cancer.gov/causespecific/, and https://seer.cancer.gov/seerstat/.
