# Supplementary figures and images for: Risk of cardiac-related death in astrocytoma patients treated with chemotherapy: A competing risk analysis using the SEER database
Source: Front Cardiovasc Med. 2023 Apr 25;10:996354. doi: 10.3389/fcvm.2023.996354 (PMC10167282; doi:10.3389/fcvm.2023.996354)

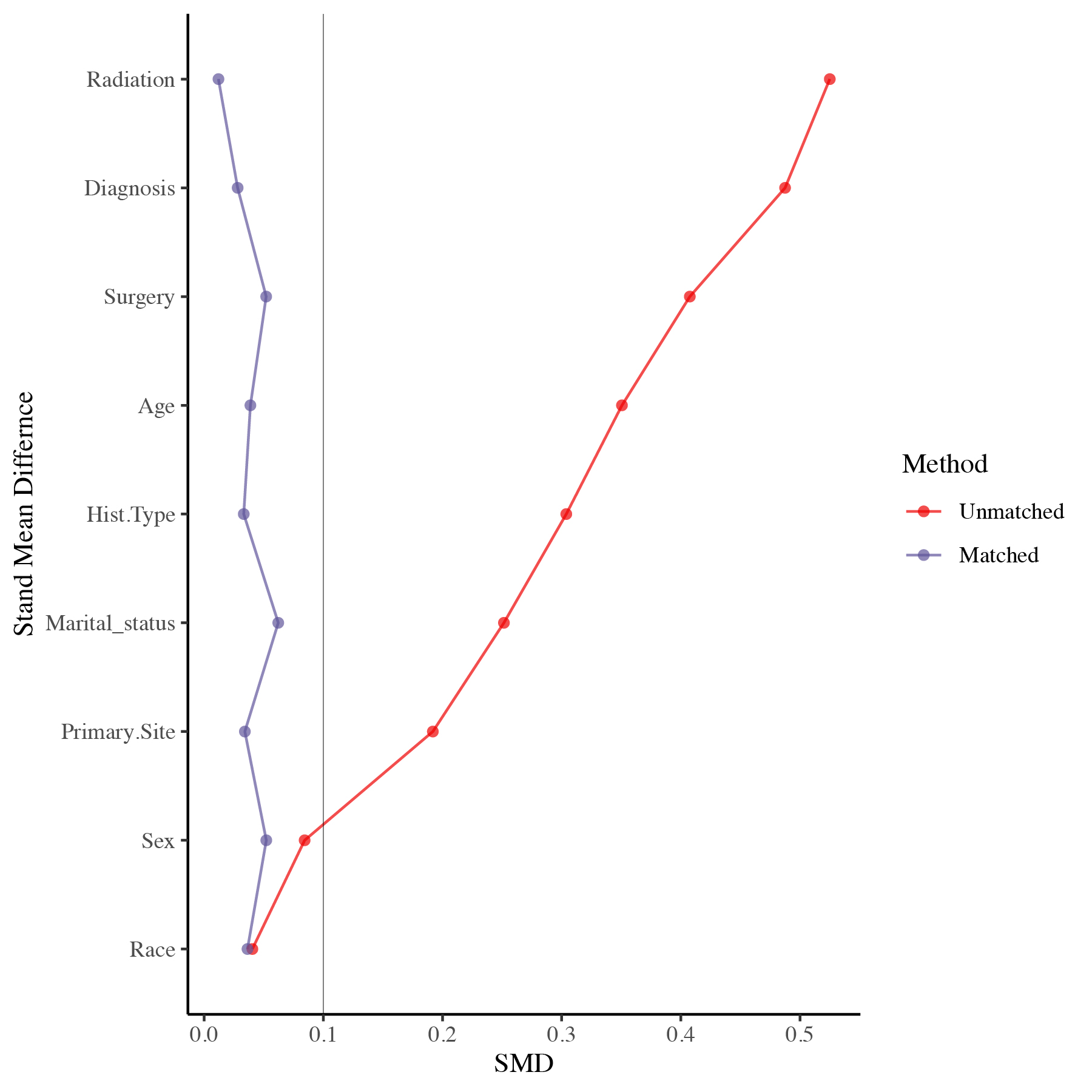

Supplement: Supplementary file 1 [file Image1.jpeg]

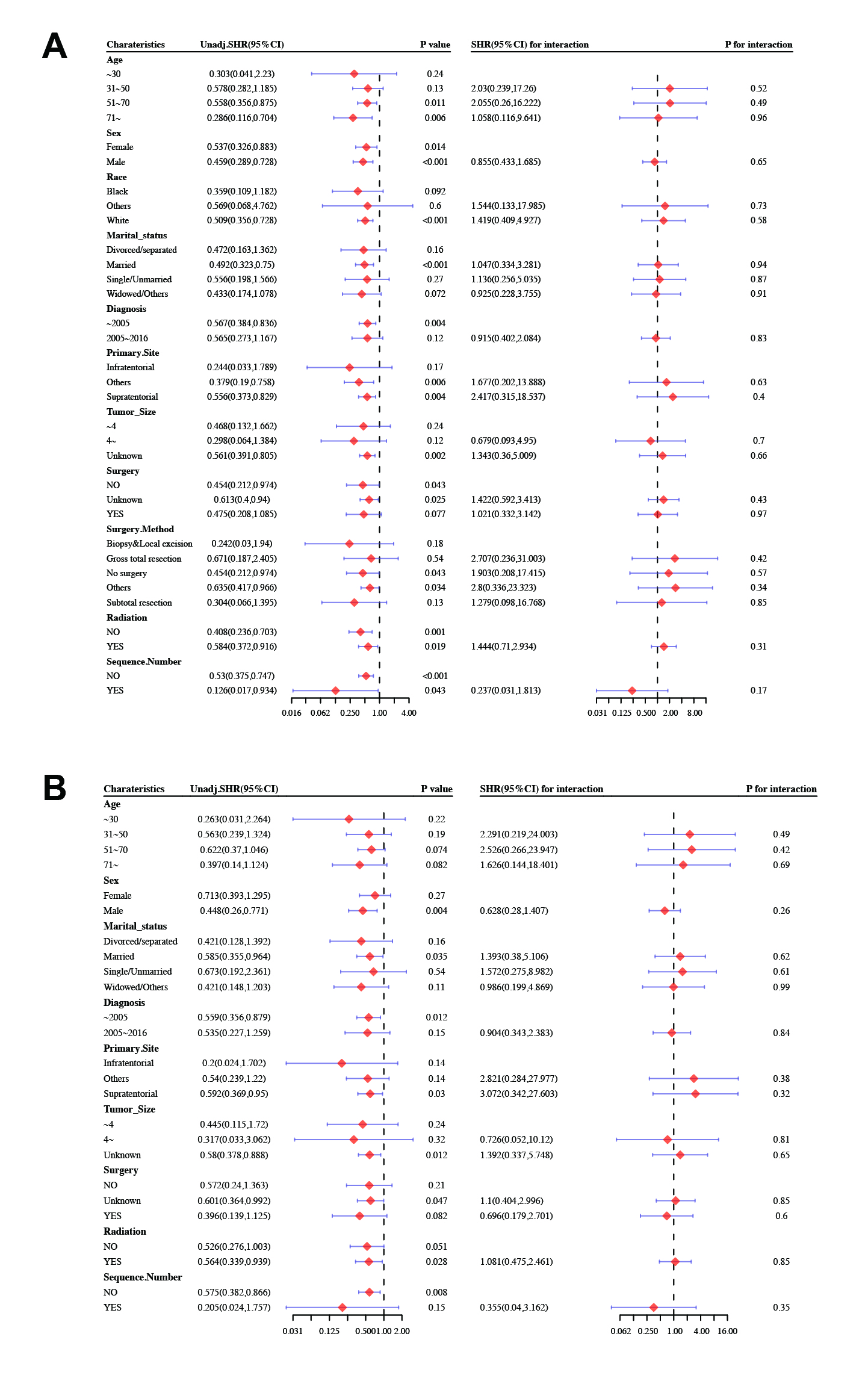

Supplement: Supplementary file 2 [file Image2.jpeg]

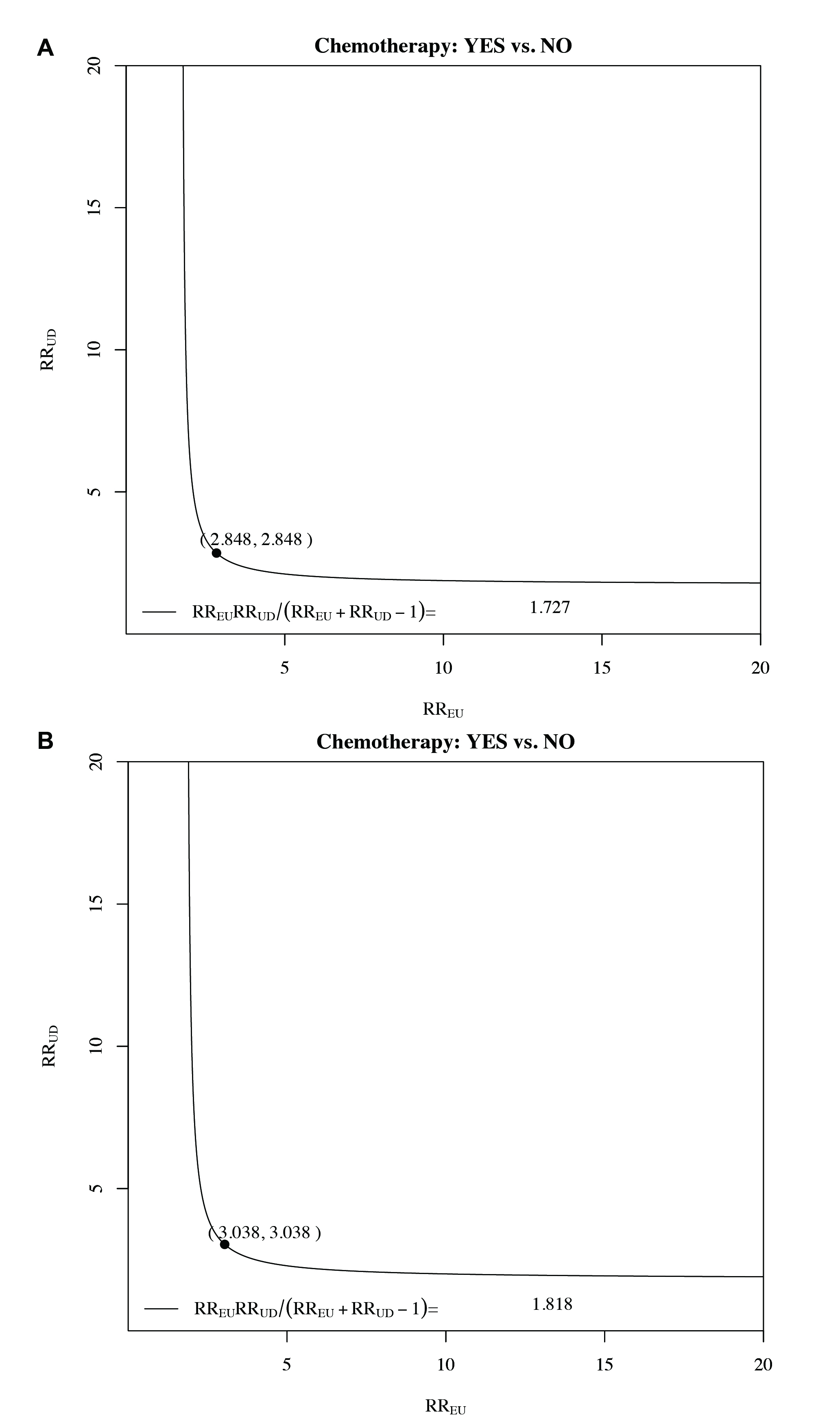

Supplement: Supplementary file 3 [file Image3.jpeg]
